# BNT162b2 Vaccination during Pregnancy Protects Both the Mother and Infant: Anti-SARS-CoV-2 S Antibodies Persistently Positive in an Infant at 6 Months of Age

**DOI:** 10.1155/2021/6901131

**Published:** 2021-10-12

**Authors:** Chetna Mangat, Natasa Milosavljevic

**Affiliations:** Department of Pediatric and Adolescent Medicine, Mayo Clinic Health System, Eau Claire, WI, USA

## Abstract

Vaccinations are the most important intervention for controlling the ongoing coronavirus disease (COVID-19) pandemic, caused by the SARS-CoV-2 virus. BNT162b2 is an mRNA-based vaccine, which is promising and safe for use during pregnancy, as it could help prevent SARS-CoV-2 infection and its complications during pregnancy. Other vaccines, such as influenza and Tdap (tetanus toxoid, reduced diphtheria toxoid, and acellular pertussis) vaccines, provide significant protection for babies. Recent studies have shown that COVID-19 antibodies are present in newborns at birth, owing to maternal BNT162b2 vaccination during pregnancy; however, it is currently unclear how long these antibodies could protect infants from SARS-CoV-2 infection and its complications. Herein, we present the case of a preterm baby born at 33 weeks via an emergency cesarean section owing to maternal complications. The mother had received two doses of the BNT162b2 vaccine at 22 and 26 weeks of gestation. Positive anti-SARS-CoV-2 S antibodies were detected in the infant at 2 weeks, 6 weeks, 3 months, and 6 months of age. This is the first case report in which BNT162b2 vaccination during pregnancy yielded a persistent immune response in an infant at 6 months of age. The declining anti-SARS-CoV-2 S antibody titers noted at 6 months of age emphasize the need for the vaccination of children at this age.

## 1. Introduction

Severe acute respiratory syndrome coronavirus SARS-CoV-2 (COVID-19) is a highly infectious respiratory virus that was first detected in Wuhan in late 2019 [1, 2]. Shortly thereafter, SARS-CoV-2 infection (COVID-19) emerged as a worldwide public health emergency that has progressed to a global pandemic and has affected millions of individuals [3, 4]. Initially, COVID-19 was thought to be a serious illness in older populations or individuals with disabilities; however, the shifting age distribution and emerging new variants indicate the increasing risk to younger age groups [5–8]. Over the last year, many vaccines have been developed to control the COVID-19 pandemic, three of which received Emergency Use Authorization (EUA) in the USA [9–11]. On August 23, 2021, Pfizer-BioNTech COVID-19 vaccine received formal approval from the Food and Drug Administration (FDA) to be used for the prevention of SARS-CoV-2 infection in individuals aged 16 years and above [12]. Vaccines were rolled in a phasic manner per risk stratification and are currently approved for use in individuals aged 12 years and above [13]. However, with the rapidly evolving pandemic and the emergence of new variants, it is important to protect all age groups to halt this pandemic [14, 15]. Early data from vaccine trials in children are promising with regard to the safety and efficacy of these vaccines in younger children, which could play a major role in controlling this pandemic [16, 17].

For many years, influenza and Tdap (tetanus toxoid, reduced diphtheria toxoid, and acellular pertussis) vaccines have been recommended for use during pregnancy to protect newborns. They are found to be safe during pregnancy and protect newborns via passage of antibodies through the placenta [18–20].

BNT162b2 vaccine is a new vaccine, and based on available data, it is safe for use in pregnant women [21]. According to the Centers for Disease Control and Prevention (CDC) and American College of Obstetricians and Gynecologists (ACOG), any approved COVID-19 vaccine can be used during pregnancy and lactation [22, 23]. Vaccination during pregnancy not only helps protecting from COVID-19 and its complication in the mother but could also protect the infant from the infection by passive transfer of antibodies. We present a case in which maternal BNT162b2 vaccine resulted in positive anti-SARS-CoV-2 S antibodies in an infant beyond neonatal period.

## 2. Case Presentation

A preterm baby was born at 33 weeks of gestation via emergency cesarean section because of nonassuring fetal heart tones. During labor, the mother presented with nausea and vomiting with impending diabetic ketoacidosis; at the time of admission, she was swabbed for SARS-CoV-2 PCR testing as per the hospital policy, and the test result was negative. The baby required neonatal resuscitation in the delivery room, which included stimulation, positive pressure ventilation, and continuous positive airway pressure. She developed mild respiratory distress syndrome due to premature gestational age and maternal complications. She was then intubated and required surfactant treatment and was transferred to the neonatal intensive care unit by a neonatal transfer team. On day 2 of life, she transitioned to room air. On day 12, she was transferred back to a local special care nursery to work on her oral feeds.

The maternal complications included hyperemesis gravidarum and type I diabetes mellitus. The mother is a healthcare worker and received the first dose of BNT162b2 vaccine at 22 weeks and the second dose at 26 weeks of gestation.

### 2.1. Diagnostic Assessment

The mother requested a serological test on her baby to determine whether she transferred some antibodies from the vaccination during pregnancy. Antibodies against SARS-CoV-2 spike protein (anti-SARS-CoV-2 S) were tested in the baby at two weeks of life, and the spike titer was >250 U/mL. The test was performed at the Mayo Clinic Laboratory using the Roche Elecsys Anti-SARS-CoV-2 S Reagent [24]; this test is a semiquantitative test that has received EUA from the FDA [25]. The test results are interpreted as negative for anti-SAR-CoV-2 S if the titer is < 0.80 U/mL but positive if the titer is 0.80–250 U/mL or >250 U/ml. An electrochemiluminescence immunoassay test with a modified double-antigen sandwich technique, which is a validated immunoassay test, was used for the qualitative and semiquantitative detection of the antibodies against the SARS-CoV-2 spike (S) protein receptor-binding domain in human serum and plasma, which predominately detects IgG antibodies [26, 27].

The baby had an immature feeding pattern secondary to prematurity, but which resolved over time. She did not develop any other complications related to prematurity and was discharged home at 3 weeks of age in a relatively good condition.

### 2.2. Follow-Up

Anti-SARS-CoV-2 S antibodies were tested again at 6 weeks, 3 months, and 6 months of life. The titers were >250 U/mL at 6 weeks and 3 months of life; however, the titers decreased to >35 U/mL at 6 months of life (Table 1).

Anti-SARS-CoV-2 S antibodies could be positive after vaccination or after infection, but anti-SARS-CoV-2 nucleocapsid (N) antibodies are specific to recent or past infection. SARS-CoV-2 nucleocapsid (N) antibodies were negative in the infant at 6 weeks, 3 months, and 6 months of age (Table 1). The negative results for the antibodies to nucleocapsid (N) antigen suggested that the immune response in the infant is attributed to maternal immunization rather than to a past infection in the mother or infant. This qualitative test was performed at the Mayo Clinic Laboratory using the Roche Elecsys Anti-SARS-CoV-2 Reagent assay from Roche Diagnostics using recombinant protein representing the nucleocapsid (N) antigen for the determination of antibodies against SARS-CoV-2 [27, 28].

## 3. Discussion

Vaccinating pregnant women to protect mothers, fetuses, and infants from infection has increasingly been performed over the last decade. It offers an opportunity to provide protection to immunologically immature infants before they can receive their own vaccinations. Vaccines against tetanus, pertussis, and influenza are recommended for use during pregnancy [18–20]. Maternal antibodies are transferred from the mother to the infant during pregnancy via transplacental transport. The placental transport system is highly selective for IgG antibodies and essentially excludes the transport of other major immunoglobulin classes, including IgE, IgM, and IgA. Transplacental transport is regulated by the neonatal Fc receptor which is a highly complex process. Most studied factors that may modulate placental transfer are the IgG subclass, glycosylation of antibody, total maternal IgG concentration, maternal disease, infant gestational age, and birth weight. The transfer of antibodies is minimal before 16 weeks but increases throughout the second trimester and peaks in the third trimester, especially in the last 4 weeks of pregnancy, which could lead to higher antibody titers in the baby than in the mother [29–31]. This process is complex and is not fully understood. It could be attributed to the increased cytotrophic expression in early expression, consequently interfering with antibody transfer; another hypothesis is that the neonatal Fc receptor is highly expressed with advancing gestation, and premature infants may experience insufficient antibody transfer through the placenta [32]. However, premature infants born at 28–35 gestational weeks to mothers vaccinated during pregnancy with Tdap or inactivated polio vaccine were found to have significantly higher antibody concentrations at 2 months of age than infants born at the same gestational age to unvaccinated mothers [33]. In our case, the mother received BNT162b2 vaccine at 22 and 26 weeks of gestational age, and the infant was born at 33 weeks of gestational age but still retained positive anti-SARS-CoV-2 S antibody titers at 6 months of age. This indicates that BNT162b2 vaccines given in the second trimester could still provide significant protection to preterm infants; however, term infants could receive greater benefits from maternal vaccination, as the antibody transfer increases with advancing gestational age.

Pregnant women with COVID-19 are at an increased risk for severe illness as compared with nonpregnant women of reproductive age [34]. In addition, they might be at an increased risk for adverse pregnancy outcomes, such as preterm birth, as compared with pregnant women without COVID-19 [35]. The CDC and Advisory Committee on Immunization Practices (ACIP), in collaboration with the ACOG and the American Academy of Pediatrics, have issued guidelines indicating that approved COVID-19 vaccines can be administered to pregnant women [22, 23]. The preliminary findings of the V-safe After Vaccination Health Checker surveillance system, v-safe pregnancy registry, and Vaccine Adverse Event Reporting System aimed to study the initial safety of mRNA COVID-19 vaccines in pregnant women did not show any obvious safety concerns [21].

Emerging research has shown some evidence of transplacental transfer of anti-SARS-CoV-2 S antibodies after maternal mRNA COVID-19 vaccination and suggests that maternal vaccination might provide some level of protection to the infant at birth [36–38]. A recent study showed the neutralizing effect of these antibodies; however, at this time, it is unclear how long these protective antibodies will be present in the baby [39].

In this unique case, the mother received BNT162b2 vaccine during her second trimester at 22 and 26 weeks, and the baby was born preterm at 33 weeks of gestation secondary to maternal complications. We observed persistence of anti-SARS-CoV-2 S antibody titers in the infant at 2 weeks, 6 weeks, 3 months, and 6 months of age. However, the titers dropped to >35 U/mL at 6 months of age as compared with titers of >250 U/mL at 2 weeks, 6 weeks, and 3 months of age.

This healthy baby is growing and developing well and did not develop any complications related to premature age. Initially, she was breastfed and required supplemental formula feeding; currently, she is receiving donor breast milk and a 22-calorie formula per maternal preference.

This report is based on only one case; thus, more extensive research on this topic is needed to determine the role of maternal vaccination in preventing COVID-19 and its complications in infants.

### 3.1. Conclusions

Herein, we report the first case in which persistence of anti-SARS-CoV-2 S antibodies was noted in a preterm infant at 6 months of age, which correlates with the prevention of COVID-19 and its complications in early infancy.

Thus, vaccinating pregnant women with BNT162b2 vaccine not only protects the mother but also the infant via transfer of anti-SARS-CoV-2 S antibodies. In our case report, we also observed that titers were low at 6 months of age compared with the titers at 2 weeks, 6 weeks, and 3 months of age. This emphasizes the need for starting vaccination against COVID-19 at 6 months of age. Based on these findings, extensive research is required to determine whether pregnant women require booster vaccinations, as significantly high titers of anti-SARS-CoV-2 S antibodies can be passed to their infants, which could protect them against COVID-19 and its complications at birth and early infancy.

## Figures and Tables

**Table 1:** Serial titers of anti-SARS-CoV-2 S antibodies and persistently negative serology to nucleocapsid (N) antigen.

	Age of the infant			
SARS-CoV-2 serology in the infant	2 weeks	6 weeks	3 months	6 months
SARS-CoV-2 nucleocapsid total Ab, S	Not tested	Negative	Negative	Negative
SARS-CoV-2 spike Ab, S	Positive	Positive	Positive	Positive
SARS-CoV-2 spike Ab, quant, S U/ml	>250	>250	>250	>35

## Data Availability

The data is available in the form of serial antibody titers, which can be shared if requested.
